# 13-Year-Old Boy Presenting with Bilateral Femur Fractures in the Setting of Severe Vitamin D Deficiency

**DOI:** 10.1155/2021/2440999

**Published:** 2021-08-04

**Authors:** Marianne Jacob, Marisa Censani

**Affiliations:** Weill Cornell Medicine, New York Presbyterian Hospital, Division of Pediatric Endocrinology, Department of Pediatrics, New York, NY, USA

## Abstract

**Background:**

Femur fractures in adolescents are rare. Severe vitamin D deficiency has important implications for bone health. We describe the case of a 13-year-old boy with autism spectrum disorder (ASD) who presented with low-impact bilateral femur fractures in the setting of severe vitamin D deficiency. *Case Presentation*. A 13-year-old boy with ASD presented with bilateral leg pain after an unwitnessed fall. Laboratory investigations revealed severe hypocalcemia (S. calcium 4.9 mg/dL) and severe vitamin D deficiency (25(OH)D < 4 ng/mL). Lower extremity X-rays revealed bilateral distal femoral metaphyseal fractures.

**Conclusion:**

This is the youngest known case of bilateral femoral fractures in the setting of severe 25(OH)D deficiency. Children with ASD are especially at risk for 25(OH)D deficiency as many have inadequate nutritional intake. As such, primary care providers may provide a pivotal role in the routine laboratory screening of 25(OH)D in this population.

## 1. Introduction

Vitamin D (25(OH)D) deficiency has important implications for bone health. Decreased exposure to sunlight, a major source of vitamin D, has become an important factor in the development of vitamin D deficiency, especially as children spend much time indoors. Individuals with darker skin tones have also been found to require longer sun exposure in order to synthesize cholecalciferol and are at greater risk for deficiency as well [1, 2]. Few foods are fortified with vitamin D, and therefore, deficiency may not be secondary to poor nutrition alone. Severe 25(OH)D deficiency has the potential to present with significant osteopenia and low-impact fracture. Low-impact fracture has been defined as a fracture occurring from a fall no greater than standing height or occurring spontaneously [3]. Here, we present the youngest reported case of low-impact bilateral femur fractures in the setting of severe 25(OH)D deficiency.

## 2. Case Presentation

**Figure 1:**
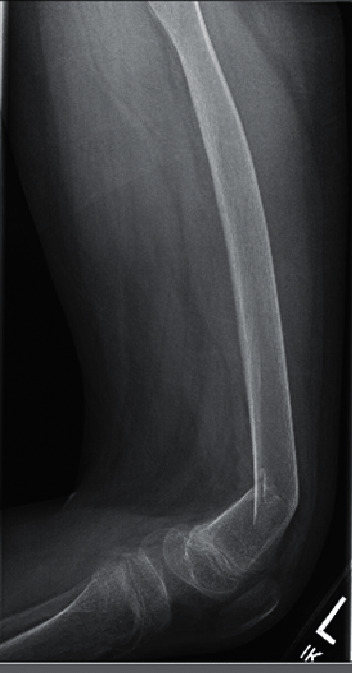
Left femur fracture.

**Figure 2:**
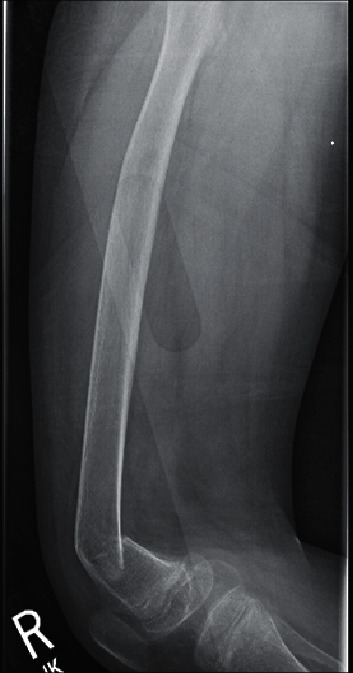
Right femur fracture.

A 13-year-old boy presented to the pediatric emergency department with bilateral leg pain after an unwitnessed fall onto the street from the sidewalk. There was no history of major trauma, syncope, or seizure. He had severe autism spectrum disorder (ASD) with poor verbal skills. On examination, he was crying and had tenderness over the bilateral knees and right ankle. The range of motion of the hip and knee was limited by pain. There were no bony deformities. Lower extremity X-rays revealed acute comminuted and impacted fractures of the bilateral distal femoral metaphyses with anterolateral apical angulation of the distal fracture fragments (Figures 1 and 2). Diffuse osteopenia was noted, but no other radiographic signs of rickets were seen, including metaphyseal widening, splaying, cupping, fraying, or prominence of the costochondral junctions on chest X-ray.

On physical examination, his height was at the 4^th^ percentile (appropriate with his midparental target height at the 10^th^ percentile). His weight was at the 52^nd^ percentile and his body mass index (BMI) at the 87^th^ percentile, meeting the criteria for overweight. His physical exam, apart from his limited lower extremity movement, was remarkable for a positive Chvostek sign, early Tanner stage 3 development with 8 cc testes descended bilaterally, and vertical fingernail ridges. Skin examination was negative for bruising, acanthosis, and striae. The differential diagnosis included vitamin D deficiency, malabsorptive disorder (i.e., celiac disease or inflammatory bowel disease), osteogenesis imperfecta, prolonged steroid use, renal failure, hypothyroidism, and juvenile osteoporosis.

Initial laboratory investigations revealed a serum calcium of 4.9 mg/dL with serum albumin of 4.4 g/dL. An ionized calcium of 0.61 mmol/L confirmed hypocalcemia. Parathyroid hormone (PTH) was elevated at 280.7 pg/mL and there was evidence of severe deficiency in both 25(OH)D (<4 ng/mL) and 1,25(OH)2D (17.8 pg/mL). Nutritional labs revealed a low vitamin A, vitamin B12, vitamin B6, and zinc as described in Table 1. There were notable coagulopathies, with deficiencies in factors II (73%), V (27%), and VII (37%). Other labs revealed an elevated alkaline phosphatase of 290 ug/L and normal thyroid function with a normal thyroid-stimulating hormone (TSH) of 1.137 uIU/mL, free thyroxine of 0.8 ng/dL, and total thyroxine of 6.1 ug/dL. Renal function showed normal creatinine (0.5 mg/dL) and blood urea nitrogen (9.0 mg/dL). Erythrocyte sedimentation rate was normal (<1 mm/hr) and a celiac screen was negative (tissue transglutaminase IgA antibody <2 U/mL with normal IgA of 120 mg/dL). Urine calcium-to-creatinine ratio was low at 0.01 mg/dL. An electrocardiogram revealed a prolonged QTc of 498 milliseconds.

The patient did not have a history of apparent numbness, weakness, muscle cramps, or bone pain. In the prior few months, however, he was noted to go down steps with two feet, one step at a time, and this had been a change in behavior. He was raised in the Northeast United States in New York and was of Hispanic ethnicity. His mother described him as a picky eater. On further dietary recall, he was noted to eat potato chips, French fries, corn, crackers, pizza without cheese, and chicken nuggets. He mainly drank ginger ale and apple juice. His lifestyle was sedentary with most time spent indoors or riding on the train. There was no personal or family history of metabolic bone disease. He had no history of prolonged steroid use.

Given his hypocalcemia, treatment was initiated with intravenous calcium gluconate 2 grams every 2-3 hours with a goal total calcium of greater than 8 mg/dL so that he would be stable for orthopedic surgery. After 48 hours of intravenous calcium treatment, he underwent bilateral antegrade nailing of the bilateral femurs. He was started on vitamin D3 50,000 international units once weekly. Postoperatively, enteral calcium carbonate of 100 mg/kg/day divided every 6 hours was ordered. However, due to his refusal to take enteral calcium, a nasogastric tube was placed and his dose was increased to 120 mg/kg/day divided every 8 hours. Total calcium levels continued to improve and enteral calcium was slowly weaned to 60 mg/kg/day divided twice daily. By the day of discharge, his calcium level had improved to 9.9 mg/dL and 25(OH)D concentration improved to 13 ng/mL. He was discharged to a rehabilitation center for further feeding therapy and physical therapy.

## 3. Discussion

Vitamin D is a key regulator in calcium and phosphate homeostasis, aiding in normal bone mineralization. 25(OH)D deficiency therefore has important implications for overall bone health. As 25(OH)D deficiency has increased in prevalence in children and adolescents worldwide [4, 5], severe vitamin D deficiency (25(OH)D < 12 ng/dL) has also become more prevalent with one study reporting an increase from 3 to 8% in 12- to 15-year-olds from 1988–1994 to 2001–2006 [6]. This degree of deficiency can lead to serious sequelae, such as low bone density and fracture [7]. Therefore, action has been taken towards prevention.

Osteopenia is one of the earliest radiographic signs of 25(OH)D deficiency. However, as the deficiency persists, signs of rickets can be noted, such as metaphyseal widening and fraying [8]. Moreover, the risk for fracture increases as such bony changes are identified. Imaging findings in our patient revealed diffuse osteopenia, which likely contributed to his bilateral distal femoral metaphyseal fractures.

Few cases have described bilateral femur fractures in children with moderate to severe 25(OH)D deficiency. There have been reports of older adolescents with severe vitamin D deficiency who presented in the setting of hypocalcemic seizure. Schnadower et al. [9] described the case of a 17-year-old male child who had a first-time seizure resulting in bilateral femoral neck fractures. Laboratory workup revealed a calcium level of 4.5 mg/dL and 25(OH)D of <5 ng/mL. Similarly, another case described a 16-year-old male child with hypocalcemic seizure (6.8 mg/dL) secondary to 25(OH)D deficiency of 8 ng/mL. He, too, developed bilateral femoral neck fractures [10]. Adolescent boys are found to have a higher rate of bone fracture as compared to girls, with a peak incidence between the ages of 13 and 14 years [11]. Fracture risk is also related to rapid pubertal growth—a period of increased cortical weakness [12]. Based on prior growth charts and exam, our patient appeared to have had an increased growth velocity of 9.7 cm/year (annualized over 10 months) and 8 cc testes on the exam, indicative of pubertal progression and growth.

Interestingly, our patient developed femoral fractures, which are rare as compared to the more common distal forearm fractures seen in adolescence [11]. Although fractures more commonly occur at the femoral shaft (64%), our patient's fracture was in a distal femoral location (27%) [13]. With prior reports of bilateral femur fractures in the setting of hypocalcemic seizure, proximal fractures may be more common due to the powerful nature of the violent contractions of the proximal thigh [14]. Our patient's distal femoral fractures may have been related to significant osteopenia secondary to severe 25(OH)D deficiency. Dual-energy X-ray absorptiometry (DXA) scan, although useful for diagnostic purposes, was deferred due to the urgency of surgical management.

A history of ASD likely contributed to our patient's degree and presentation of 25(OH)D deficiency. Children with ASD are at increased risk for nutritional deficiencies, including vitamin D, given their food selectivity, often severely restricting their diet to up to a few foods [15]. Factors such as texture and appearance highly influence food selectivity [16], and factors such as brand, product name, and packaging also play a role in food choice [17]. Our patient notably restricted himself to a few foods that were of similar color (yellow) and had strong preferences for specific brands.

As children with ASD have varying degrees of verbal skills [18], it can also be difficult for the caregiver to recognize signs and symptoms of hypocalcemia such as perioral numbness, tingling, and/or muscle cramps. His mother did report that he had a peculiar way of walking down the stairs for several months, which may have been a presentation of hypocalcemia or stress fracture. As such, behavioral changes should be considered as a sign of hypocalcemia or fracture in children with ASD suspected to have 25(OH)D deficiency.

## 4. Conclusions

To our knowledge, this is the youngest reported case of bilateral femur fractures in the setting of severe 25(OH)D deficiency. When an adolescent presents with low-impact femur fractures, 25(OH)D deficiency should be considered. Clinicians should recognize that children with ASD are at higher risk for vitamin and mineral deficiencies and require close nutritional evaluation for adequate calcium and vitamin D intake. Primary care providers may therefore provide a pivotal role in the routine laboratory screening of 25(OH)D in this population.

## Figures and Tables

**Table 1:** Initial laboratory tests performed.

Lab	Result	Normal range
Calcium	4.9 mg/dL	8.4–10.2 mg/dL
Albumin	4.4 g/dL	3.2–5.0 g/dL
Ionized calcium	0.61 mmol/L	1.12–1.32 mmol/L
Urine calcium : creatinine	0.01 mg/dL	<0.15 (<0.2)
Vitamin D25	<4 ng/mL	30.0–80.0 ng/mL
Vitamin D 1,25	17.8 pg/mL	19.9–79.3 pg/mL
Parathyroid hormone	280.7 pg/mL	18.0–88.0 pg/mL
Alkaline phosphatase	290 ug/L	27.8–210.0 ug/L
Phosphorous	4.6 mg/dL	2.3–5.4 mg/dL
Magnesium	2.0 mg/dL	1.6–2.6 mg/dL
Vitamin A	0.13 mg/dL	0.26–0.70 mg/L
Vitamin B12	124 pg/mL	211–911 pg/mL
Vitamin B6	8.6 nmol/L	20.0–125.0 nmol/L
Vitamin C	71 umol/L	23–114 umol/L
Vitamin E	7.7 mg/L	5.5–18.0 mg/L
Zinc	31.2 ug/dL	60.0–120.0 ug/dL
TSH	1.137 uIU/mL	0.550–4.780 uIU/mL
Free thyroxine	0.8 ng/dL	0.9–1.8 ng/dL
Total thyroxine	6.1 ug/dL	4.5–10.9 ug/dL
Tissue transglutaminase IgA	<2 U/mL	0–3 U/mL
Immunoglobulin A	120 mg/dL	58–358 mg/dL
Prothrombin time	20.1 seconds	12.1–15.4 seconds
INR	1.6	0.8–1.3
Activated PTT	33.4 seconds	23.0–37.0 seconds
Factor II	73%	79–131%
Factor V	27%	562–139%
Factor VII	37%	50–129%
Factor VIII	233%	50–150%
Factor IX	72%	65–150%

## Data Availability

All the data supporting this article are included in the article itself.
